# Anti-idiotype isolation of a broad and potent influenza A virus-neutralizing human antibody

**DOI:** 10.3389/fimmu.2024.1399960

**Published:** 2024-05-30

**Authors:** Adam S. Olia, Madhu Prabhakaran, Darcy R. Harris, Crystal Sao-Fong Cheung, Rebecca A. Gillespie, Jason Gorman, Abigayle Hoover, Nicholas C. Morano, Amine Ourahmane, Abhinaya Srikanth, Shuishu Wang, Weiwei Wu, Tongqing Zhou, Sarah F. Andrews, Masaru Kanekiyo, Lawrence Shapiro, Adrian B. McDermott, Peter D. Kwong

**Affiliations:** ^1^ Vaccine Research Center, National Institute of Allergy and Infectious Diseases, National Institutes of Health, Bethesda, MD, United States; ^2^ Division of Viral Products, Center for Biologics Evaluation and Research, Food and Drug Administration, Silver Spring, MD, United States; ^3^ Department of Biochemistry and Molecular Biophysics, Columbia University, New York, NY, United States; ^4^ Aaron Diamond AIDS Research Center, Columbia University, Vagelos College of Physicians and Surgeons, New York, NY, United States; ^5^ Zuckerman Mind Brain Behavior Institute, Columbia University, New York, NY, United States

**Keywords:** anti-idiotype, broadly neutralizing antibody, cryo-EM, influenza A virus, MEDI8852, VH6-1 class

## Abstract

The VH6-1 class of antibodies includes some of the broadest and most potent antibodies that neutralize influenza A virus. Here, we elicit and isolate anti-idiotype antibodies against germline versions of VH6-1 antibodies, use these to sort human leukocytes, and isolate a new VH6-1-class member, antibody L5A7, which potently neutralized diverse group 1 and group 2 influenza A strains. While its heavy chain derived from the canonical IGHV6-1 heavy chain gene used by the class, L5A7 utilized a light chain gene, IGKV1-9, which had not been previously observed in other VH6-1-class antibodies. The cryo-EM structure of L5A7 in complex with Indonesia 2005 hemagglutinin revealed a nearly identical binding mode to other VH6-1-class members. The structure of L5A7 bound to the isolating anti-idiotype antibody, 28H6E11, revealed a shared surface for binding anti-idiotype and hemagglutinin that included two critical L5A7 regions: an FG motif in the third heavy chain-complementary determining region (CDR H3) and the CDR L1 loop. Surprisingly, the chemistries of L5A7 interactions with hemagglutinin and with anti-idiotype were substantially different. Overall, we demonstrate anti-idiotype-based isolation of a broad and potent influenza A virus-neutralizing antibody, revealing that anti-idiotypic selection of antibodies can involve features other than chemical mimicry of the target antigen.

## 1 Introduction

Influenza A virus causes seasonal respiratory illnesses that are associated with a substantial clinical burden globally, resulting in up to 650,000 deaths each year (https://www.who.int/news-room/fact-sheets/detail/influenza-(seasonal)). In addition, influenza A virus has the potential to cause pandemics, and pandemic strains of the virus can cause severe diseases with high fatality rates (1–4). Influenza vaccines are fundamental to disease prevention. Current vaccination strategies require annual vaccine reformulations based on virus types predicted to be most common in the upcoming influenza season (5). This approach has, however, been problematic with vaccine efficacy as low as 10% in some years, owing to discordance between predicted and circulating strains (6, 7). Vaccines or antivirals that can offer “universal” protection against a diverse array of influenza strains are needed to prevent future flu pandemics.

Broadly neutralizing antibodies (bNabs) represent a promising avenue for universal protection against influenza virus. Hemagglutinin (HA) is the major surface protein on the influenza virus and comprises a less-conserved immunodominant head domain and a relatively more-conserved immuno-subdominant stalk domain. Several bNabs, such as MEDI8852 (8, 9), FI6 (10), and CR9114 (11), that target the conserved stalk domain have been identified and are cross-reactive and protective in animal models against diverse influenza strains. Such antibodies have the potential for clinical development as therapeutic agents to treat severe influenza cases, including zoonotic influenza virus infections such as H5N1 or H7N9, and as prophylactic agents for vulnerable human populations (children, immunocompromised, elderly or pregnant individuals) during pandemics. Stalk-targeting influenza bNabs administered intravenously as human IgG monoclonal antibodies have been shown to reduce influenza symptoms and viral loads (12, 13). Some of the most potent and broad anti-stalk influenza bNabs belong to the VH6-1 public class (8, 9, 14, 15). Antibodies of this class show characteristic immunoglobulin sequence features, such as the use of the IGHV6-1 gene and the presence of an “FG” motif in the third heavy chain-complementary determining region (CDR H3). Isolation and characterization of antibodies of public classes, such as the VH6-1 class, in addition to developing universal flu therapeutics, can provide insights for better understanding of population-level immune responses against influenza and guiding clonotype-specific vaccine development.

Here we investigate the use of anti-idiotype antibodies for isolation of antibodies with specific desirable features. Anti-idiotype antibodies are antibodies that bind to variable domains of other antibodies, and anti-idiotype antibodies specific for characteristic features of bNabs have been used as tools in vaccine research with potential roles hypothesized in precision medicine (16–18). For example, anti-idiotype antibody G6, which specifically recognizes IGHV1-69 F-alleles, has been used extensively to study F-allelic-based antibody and cellular responses, diseases processes, and effects of IGHV1-69 polymorphism on immunity to viruses such as influenza (19, 20). Anti-idiotype antibody iv8, selected for recognizing naïve B cells expressing five-residue light chain CDR L3s, has been used in HIV vaccine research to expand and mature rare anti-HIV bNab B cell precursors (21). In this study, we developed anti-idiotype antibodies that were selective for the VH6-1 class of influenza bNabs and used them to investigate the frequencies of VH6-1 class B cells in circulation and to isolate additional members of the VH6-1 class. We use cryo-EM to reveal the interaction surface of anti-idiotypic antibody and a newly isolated VH6-1 class member – and compare this to the interactive surface with hemagglutinin target. Overall, we use anti-idiotypic antibodies to isolate a new lineage of VH6-1 class bNabs (named L5A7), which derived from a light chain VK gene unique among known VH6-1 class antibodies, and provide insight into the ability of anti-idiotypic antibodies to select for general class features.

## 2 Methods

### 2.1 Protein expression and purification

Influenza hemagglutinin proteins were expressed as previously described (22). EXPI293 cells at 2x10^6^ cells per mL were transfected with plasmid DNA encoding the HA fused to a C-terminal thrombin site, T4 fibritin trimerization motif, and 6xHis tag using Turbo293 transfection reagent. The cells were allowed to express protein for 6 days at 37 °C, after which time the culture was centrifuged and the supernatant harvested. The supernatant was applied to His-Pure nickel resin, washed with PBS supplemented with 20 mM imidazole, and eluted with 300 mM imidazole in PBS. Following affinity purification, the HA protein was further purified by size exclusion chromatography on a Superdex S200 column pre-equilibrated in PBS. Antibody expression was performed in the same manner, and the supernatant applied to Protein A resin. The resin was washed with PBS, and antibody eluted with 20 mM glycine pH 2.0. After neutralization the antibody was dialyzed against PBS prior to storage.

### 2.2 Mouse immunization study

Murine immunization experiments were performed at GenScript, with IACUC approval number ANT17-003. Female mice (C57BL/6) were immunized every 2-weeks with 56.a.09 UCA antigen binding fragment (Fab) four times for the homologous immunizations. For the heterologous immunizations, mice were dosed with MEDI8852 UCA Fab four times, then boosted with one dose with 56.a.09 UCA Fab, followed by another boost with Fab containing 46167 UCA heavy chain and MEDI8852 UCA light chain. Two weeks later, a terminal boost was carried out with a mixture of all three Fabs. All immunization were performed with 25 µg total protein per dose, adjuvanted with Freund’s adjuvant plus Genscript’s alum-based adjuvant. Sera samples were collected either 1- or 2-weeks post each non-terminal immunization and screened by ELISA. Mouse spleens were harvested 3 days after the terminal boost and hybridomas were generated following Genscript’s standard procedure. In short, mouse spleens were ground and added to DMEM media, and centrifuged several times followed by resuspension in DMEM to wash the B lymphocytes. The B lymphocyte suspension was mixed with SP2/0 myeloma cells and fused using a BTX ECM2001 electrofusion instrument. The cell fusion mixture was then plated at a density of 2x10^6^ cells/mL onto feeder cells in a 96-well plate. Cells were incubated at 37 °C in 7% CO_2_ for 1 week, after which the supernatant was harvested for screening. Monoclonal antibodies were initially selected by screening against MEDI8852 UCA Fab in an ELISA assay.

### 2.3 VH6-1 class antibody binding to anti-idiotypes

The binding kinetics of the anti-idiotypes to 56.a.09 and MEDI8852 UCA and to mature antibodies were assessed by surface plasmon resonance on a Biacore T-200 (GE Healthcare) in the HBS-EP+ (10 mM HEPES, pH 7.4, 150 mM NaCl, 3 mM EDTA, and 0.05% surfactant P20) buffer at 25°C. The anti-idiotypes were coupled to a CM5 chip to approximately 250 RU. A 2 µM down to 2 nM serial dilution of 56.a.09 and MEDI8852 UCA and mature antibodies were flowed through at 30 µl/min for 120 s. This was followed by a dissociation phase at 30 µl/min for 600 s. The chip was regenerated with 1 M MgCl_2_, 10 mM Glycine pH 2.0 at 30 µl/min for 120 s. HBS-EP+ buffer was used to obtain blank sensorgrams. The concentration series of the antibodies were fitted globally with Biacore T200 evaluation software using a 1:1 model of binding. Plots were generated using GraphPad Prism.

### 2.4 Sample collection

Donors’ consents were waived due to anonymized donation of blood for blood donation, blood products, and research by the donors to the NIH blood bank. Donor L16 was a 66 year old male Caucasian. Donor L5 was a 48 year old female Caucasian. Peripheral blood mononuclear cells (PBMCs) were isolated by density gradient centrifugation using Ficoll-Paque Plus. Isolated PBMCs were cryopreserved at −80 °C for future usage.

### 2.5 Probe conjugation and flow cytometry staining

Biotinylated antibody Fabs were labelled with fluorescently-conjugated streptavidin as described previously (23) or using Microscale protein conjugation kits according to the manufacturer’s instructions. Briefly, biotinylated Fabs were incubated with fluorescently-labelled streptavidin at a 4:1 fluorochrome to protein molar ratio and incubated at 4 °C for 1 hour. Peripheral blood mononuclear cells were thawed and washed with R10 media (RPMI with 10% FBS and 1% penicillin-streptomycin) and PBS. Cells were stained in FACS tubes in a cocktail of antibodies (with all antibodies at a 150-fold dilution) and anti-idiotype probes, to stain cell-surface markers and B cell immunoglobulin receptors. The cocktail of anti-human antibodies included CD19 (Beckman Coulter, IM2708U), CD20 (Biolegend, 302314), CD3 (BD Biosciences, 740187), CD14 (Biolegend, 301842), CD56 (BD Biosciences, 740171), CD16 (BD Biosciences, 563830), IgD (Biolegend, 348242) and IgG (BD Biosciences, 562581). Cells were incubated for 30 minutes at 4 °C, washed twice in R10 media, resuspended in R10 media containing 7-AAD (Thermofisher Scientific, A1310) and analyzed by flow cytometry.

### 2.6 Flow cytometry analysis and B cell sorting

Lymphocytes were first gated based on cell morphology (FSC-A/SSC-A), and doublets were removed. Dead cells and non-B cells were excluded within a dump channel (CD3−/CD14−/CD16−/CD56−). Live B cells (CD19+ CD20+) subsetted as naïve (IgD+) or memory (IgG+) that were positive for anti-idiotype probes were single cell-sorted using index sorting into dry 96-well plates. Plates were stored at -80 °C until cell lysis and reverse transcription could be performed.

### 2.7 Single-cell immunoglobulin sequencing

Single-cell immunoglobulin sequencing was performed as described previously (24). Briefly, plates were removed from -80 °C and centrifuged briefly. Reverse transcription and cDNA synthesis were performed using Superscript III reverse transcriptase kit and random hexamers with the following conditions on the thermal cycler: 42 °C for 10 min, 25 °C for 10 min, 50 °C for 60 min, and 94 °C for 5 min. Immunoglobulin gene amplification was performed using the HotStarTaq Plus DNA polymerase kit using IgH, Igκ and Igλ reverse primers using the following parameters on the thermal cycler: 95 °C for 5 min and 50 cycles of 95 °C for 30 s, either 52 °C (IgH) or 58 °C (Igκ and Igλ) for 30 s and 72 °C for 55 s, followed by a final extension of 72 °C for 10 min. Once completed, amplified PCR products were indexed with unique well-specific indices and sequenced on the Illumina sequencer.

### 2.8 Antibody binding assays

Antibody binding assays to influenza HA proteins were performed using the mesoscale discovery platform as described previously (25). Briefly, streptavidin-coated 384-well plates were coated with biotinylated influenza HA proteins for 1 hour and washed. Antibodies were serially diluted and added to the coated plates. After a 1 hour incubation, plates were washed and incubated with SULFO-TAG–conjugated anti-human IgG for 1 hour. After washing, the plates were read using 1× MSD Read Buffer using an MSD SECTOR Imager 2400. Binding curves were plotted, and the area under the curve (AUC) was determined using Prism 9. HA from the following strains were tested: H7N9 A/Shanghai/02/2013, H1N1 A/California/04/2009, H3N2 A/Hongkong/1/1968 and H5N1 A/Indonesia/05/2005 (26).

### 2.9 L5A7 crystallization and structure determination

The Fab of L5A7 was generated by using HRV-3C protease to cleave the recognition site inserted in the hinge region of the antibody, and further purified by SEC with Superdex S200 16/60 chromatography column (GE) in HEPES buffer (5 mM HEPES pH7.5 and 150 mM NaCl). L5A7 Fab crystals were grown in 17.5% polyethylene glycol 4000, 0.2 M ammonium acetate, 0.1 M sodium citrate, pH 5.6. Optimized crystals were cryoprotected in well solution plus 12.5% ethylene glycol and 12.5% glycerol and flash-frozen in liquid nitrogen. Diffraction data was collected to a resolution of 1.8 Å at the SER-CAT beamline ID-22 (Advanced Photon Source, Argonne National Laboratory). The diffraction data were processed with HKL2000 suite. Phaser in Phenix was used for molecular replacement, with previously published Fabs as the initial model input. WinCoot was used to build the model, and Phenix was used to refine it. Data collection and refinement statistics are shown in **Supplementary Table 3**.

### 2.10 CryoEM structure determination

The L5A7 Fab was incubated with INDO05 HA trimer with 2-fold molar excess Fab per HA protomer. L5A7 Fab was incubated with 28H6E11 Fab at equal concentrations. For the structure of L5A7 bound to HA, volumes of 2.3 µl at concentrations of 2 mg/ml were deposited on C-flat grids (protochip.com), and the grids were vitrified using an FEI Vitrobot Mark IV with a wait time of 30 s, blot time of 3 s, and blot force of 1. Data collection was completed on a Titan Krios electron microscope with Leginon (27) with a Gatan K3 direct detection device. Exposures were collected in movie mode for 2 s with a total dose of 57.32 e–/Å^2^ for the Fab-trimer complex and 2.5 s with a total dose of 64.12 e–/Å^2^ for the anti-idiotype complex. Images were processed using CryoSPARC 3.3 (28).

For structure of L5A7 Fab bound to 28H6E11 Fab, Fabs were incubated together at equal molar ratio and a concentration of 2 mg/ml, and then adjusted to have a final concentration of 0.005% (w/v) n-Dodecyl β-D-maltoside (DDM) to prevent preferred orientation and aggregation during vitrification. Cryo-EM grids were prepared by applying 3 μL of sample to a freshly glow discharged carbon-coated copper grid (CF 1.2/1.3 300 mesh). The sample was vitrified in liquid ethane using a Vitrobot Mark IV with a wait time of 30 s, a blot time of 3 s, and a blot force of 0. Cryo-EM data were collected on a Titan Krios operating at 300 keV, equipped with a K3 detector (Gatan) operating in counting mode. Data were acquired using Leginon (29). The dose was fractionated over 50 raw frames.

Coordinates for influenza H5 HA (PDB: 4K62) (30), the L5A7 crystal structure and an AlphaFold model of 28H6E11 were used for the initial fit to the reconstructed map. This was followed by real space refinement in Phenix (31) and then iteratively adjusted with manual fitting of the coordinates using Coot (32). Geometry and map fitting were assessed through the process using Molprobity (33) and EMRinger (34). PyMOL (www.pymol.org) and ChimeraX (35) were used to generate figures.

## 3 Results

### 3.1 Anti-idiotype antibodies from mice immunized with germline versions of influenza VH6-1 class antibodies

**Figure 1:**
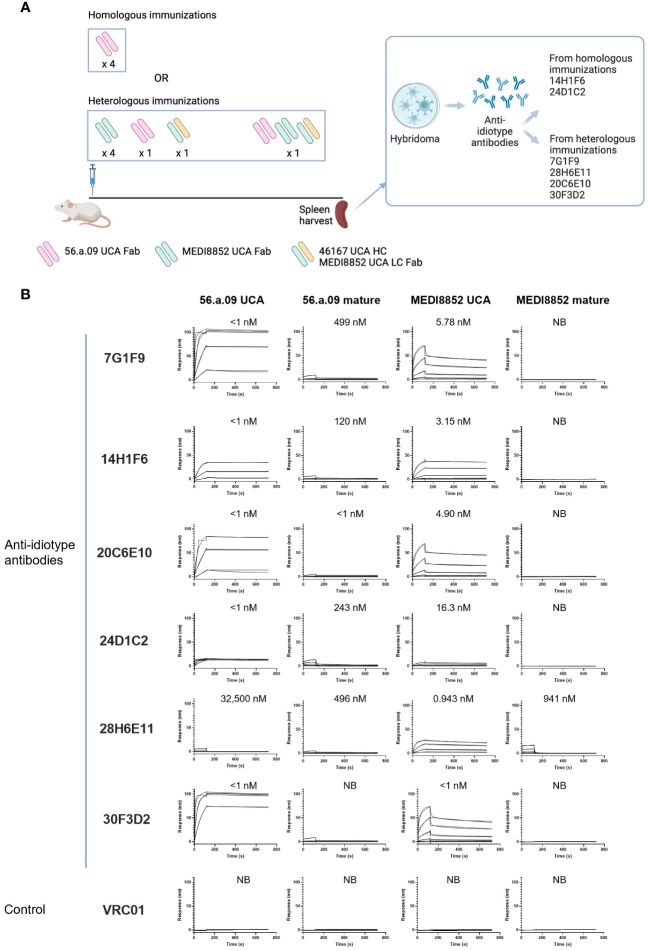
Isolation and characterization of anti-idiotype antibodies from mice immunized with the UCAs of broad VH6-1 influenza neutralizing antibodies. **(A)** Immunization scheme employing UCAs from broad VH6-1 influenza neutralizing antibodies, and work flow involving isolation of monoclonal anti-idiotype antibodies from UCA immunized mice (figure created with Biorender). **(B)** Surface plasmon resonance binding for anti-idiotype antibodies, isolated from homologous or heterologous immunized mice in **(A)** with K_D_ for each interaction shown above graph, or NB for no binding.

To obtain anti-idiotype antibodies specific for the VH6-1 class of influenza bNabs, mice were immunized with germline versions of VH6-1 class antibodies, in specific the unmutated common ancestors (UCAs) of the antigen-binding fragments (Fabs) of VH6-1 class antibodies: 56.a.09 (15), MEDI8852 (8), or a chimera of MEDI8852 and 46167 (14). Immunizations followed either a homologous regimen for which mice received four doses of only 56.a.09 UCA Fab, or a heterologous regimen for which they received six doses of UCA-Fabs from multiple VH6-1 class antibodies (**Figure 1A**). Six anti-idiotype antibodies were isolated two weeks after the final immunization using hybridoma technology, two from mice immunized with the homologous regimen and four from mice immunized with the heterologous regimen (**Figure 1A**, **Supplementary Table 1**). Surface plasmon resonance binding analysis showed that five of these six anti-idiotype antibodies bound well to either one or both 56.a.09 and MEDI8852 UCA antibodies but not to their mature counterparts (**Figure 1B**).

### 3.2 Sorting of human B cells with anti-idiotype antibodies identifies VH6-1 class antibodies

**Figure 2:**
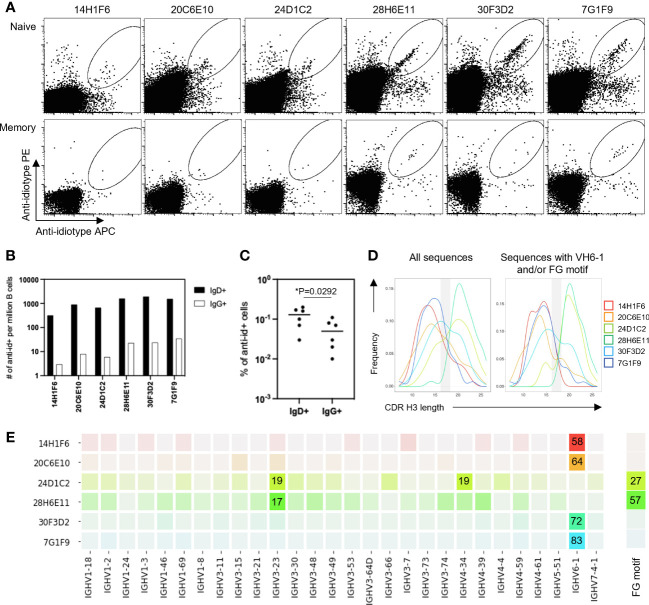
Screening of B cells from donor L16 PBMC with anti-idiotype antibodies. **(A)** Sorting of naïve and memory B cells from L16 PBMC with anti-idiotype antibodies. **(B)** Frequencies of anti-idiotype+ naïve (IgD+) and memory (lgG+) B cells isolated using the different anti-idiotype candidates. **(C)** Frequency comparison of anti-idiotype+ naïve (IgD+) and memory (IgG+) B cells. **(D)** CDR H3 length distributions of all anti-idiotype+ sequences and only sequences containing either IGHV6-1 or FG motif. **(E)** VH usage and presence of canonical "FG" motif of sequences from sorted B cells. Degree of usage is indicated by intensity of the color with more intense color indicating higher frequency. Highest frequencies are indicated in colored boxes and are shown as percentages of total number of transcripts isolated by the indicated each anti-idiotype. V-gene and FG motif percentages are calculated independently.

To determine the ability of anti-idiotype antibodies to selectively engage VH6-1 class B cells, human peripheral blood mononuclear cells (PBMCs) from donor L16 were screened against all six anti-idiotype antibodies by flow cytometry (**Figure 2A**). Anti-idiotype antibodies were conjugated to fluorochromes and used as probes. Anti-idiotype+ B cells were sorted (**Supplementary Figure 1**), and their immunoglobulin sequences obtained. On average, 90 B cells were sorted with each anti-idiotype antibody. A majority of the sorted cells comprised naïve B cells, while a relatively small proportion were from the memory compartment (**Figures 2B, C**). Paired immunoglobulin sequences were recovered from about 50% of sorted cells. Average CDR H3 lengths ranged from 12-22 amino acids for all sorted cells (**Figure 2D**). B cells sorted using four of six anti-idiotypes were enriched for IGHV6-1 gene usage (58-83% enrichment). B cells sorted using the remaining two anti-idiotypes were enriched for members with an “FG” motif in the CDR H3 region (27-57% enrichment). Both these genetic features – IGHV6-1 gene usage and the CDR H3 “FG” motif, represent sequence characteristics described for the VH6-1 class of influenza antibodies (14) (**Figure 2E**).

**Figure 3:**
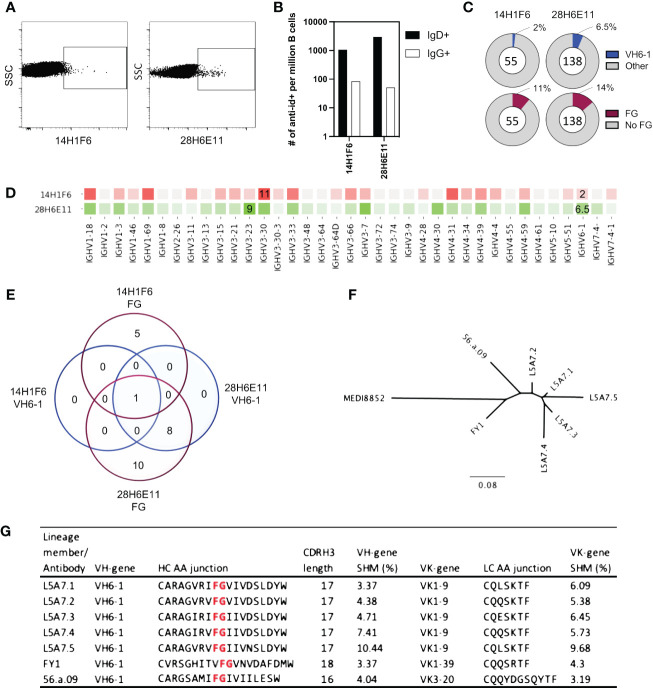
Screening of B cells from donor L5 leukopak with anti-idiotype antibodies identifies VH6-1 antibody with FG motif and different kappa chain from other members of the class. **(A)** Sorting of naïve and memory B cells from leukopak L5 with anti-idiotype antibodies. **(B)** Frequency of anti-idiotype positive naïve and memory B cells **(C)** Frequency of members with VH6-1 or FG motif in sequences isolated using anti-idiotype antibodies from leukopak L5. **(D)** VH-gene distribution of sequences isolated from leukopak L5 using anti-idiotype antibodies. **(E)** Venn diagram showing shared genetic elements among sequence members isolated using anti-idiotype antibodies. **(F)** Phylogenetic tree showing distance between L5A7 lineage members and MEDI8852. **(G)** Sequence features of L5A7 lineage members.

To isolate antibodies of the VH6-1 class of influenza bNabs, we sorted B cells with two anti-idiotype antibodies, one specific for enriching B cells with IGHV6-1 gene usage and the other specific for enriching those with “FG” motif in CDR H3. Down-selection criteria for anti-idiotype antibodies included minimal background associated with binding of anti-idiotype antibody probes to control cells while inducing good separation of probe+ events by flow cytometry. The two anti-idiotype antibodies which were used for sorting were 14H1F6, which enriched for B cells utilizing the IGHV6-1 gene, and 28H6E11, which enriched for B cells of the CDR H3 “FG” motif. Both anti-idiotypes were conjugated to fluorochromes and used to screen PBMC from a second donor (L5). Anti-idiotype+ B cells were sorted (185 cells with 14H1F6 and 199 cells with 28H6E11), and immunoglobulins sequenced (**Figure 3A**). Similar to above sorting with donor L16 leukocytes, a majority of the cells sorted were naïve B cells with a relatively small proportion sorted from the memory compartment (**Figure 3B**). Paired sequences were obtained for 55 cells sorted with 14H1F6 and 138 cells sorted with 28H6E11. In this donor, the proportion of cells that exhibited IGHV6-1 gene usage ranged from 2% (cells sorted with 14H1F6) to 5% (cells sorted with 28H6E11). Similarly, the proportion of sorted cells that contained the CDR H3 “FG” motif ranged from 11% for cells sorted with 14H1F6 to 14% for cells sorted with 28H6E11 (**Figures 3C, D**). Although the B cell enrichment observed in L16 was not observed in L5, clonal analysis revealed the presence of a common lineage (named L5A7) that was sorted with both anti-idiotypes. A total of nine pairs of heavy and light chain sequences were obtained from this lineage with five pairs of sequences representing unique lineage members. One unique lineage member was isolated using antibody 14H1F6 while all five unique lineage members were isolated using 28H6E11 (**Figure 3E**, **Supplementary Table 2**). The five unique lineage members were named as L5A7.1 to L5A7.5, such that L5A7.1 had the least somatic hypermutations on the heavy chain V-gene and L5A7.5 had the most. A phylogenetic tree of the L5A7 lineage constructed using the neighbor-joining method along with other VH6-1 class antibodies is shown in **Figure 3F**. Characteristics of the L5A7 lineage are tabulated in **Figure 3G** and shown in reference to other VH6-1 class influenza antibodies. The L5A7 lineage has sequence characteristics described for the VH6-1 class of influenza antibodies, such as the usage of the IGHV6-1 gene, 17 amino acid long CDR H3, and presence of the CDR H3 “FG” motif; however, the light chain V-gene IGKV1-9 usage is unique.

### 3.3 Binding and neutralization characteristics of L5A7 lineage antibodies

**Figure 4:**
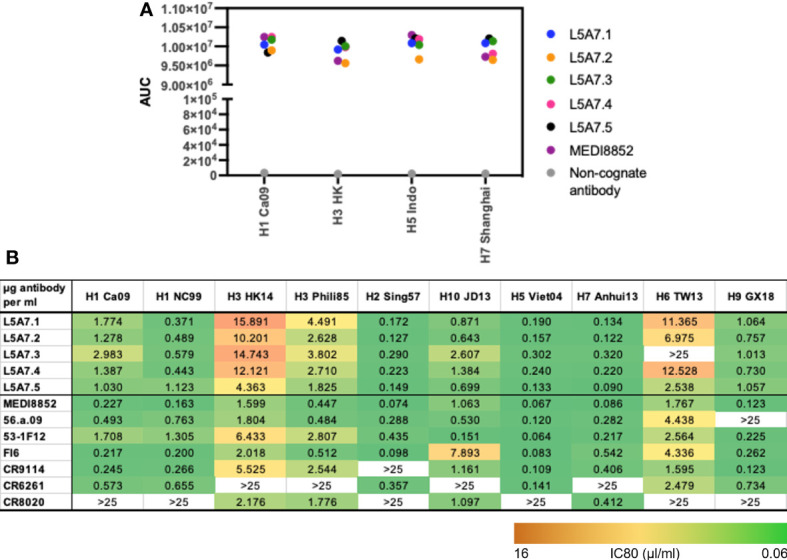
Binding and neutralization characteristics of isolated VH6-1 antibodies. **(A)** Binding of L5A7 lineage members to influenza HA shown as area under the curve (AUC). **(B)** Virus micro-neutralization indicated as IC80 titers (µg antibody per ml).

To characterize function of the L5A7 lineage, we screened lineage members for binding to recombinant hemagglutinin (HA) proteins from H1 A/California/04/2009 (Ca09), H3 A/Hong Kong/2401/2014 (HK14), H5 A/Indonesia/5/2005 (Indo05) and H7 A/Shanghai/02/2013 (Shanghai). MEDI8852 and a non-cognate antibody were screened in parallel as positive and negative controls, respectively. All L5A7 antibody lineage members showed potent binding to all four HA proteins (**Figure 4A**). L5A7 lineage members were also screened in a pseudovirus neutralization assay against H1, H2, H3, H5, H6, H7, H9 and H10 strains of influenza along with other known influenza bNabs. All L5A7 lineage members showed potent and broad neutralization. Antibody L5A7.5 was comparable to MEDI8852 in potency and breadth, neutralizing all tested strains with IC80 titers < 4.5 μg/ml (**Figure 4B**).

### 3.4 Structure of L5A7.5 bound to HA confirms VH6-1 class recognition

**Figure 5:**
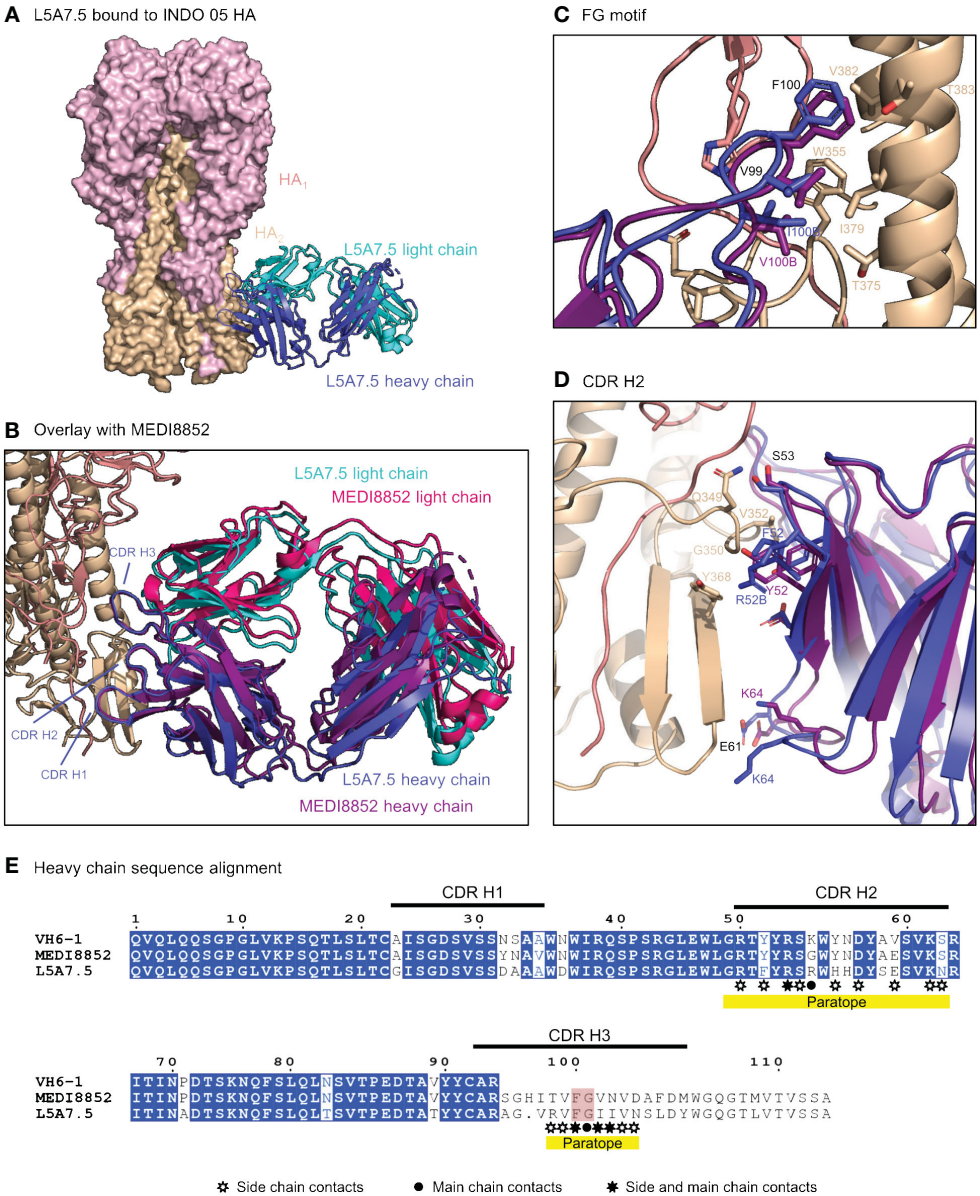
Structure of L5A7.5 bound to Flu HA shows same binding mode as MEDI8852. **(A)** Overview of L5A7.5 Fab bound to INDO05 HA. **(B)** Overlay of MEDI8852 and L5A7.5 showing near identical binding modes. **(C)** Comparison of the FG motifs and **(D)** CDR H2s between MEDI8852 and L5A7.5 with HA interacting residues shown as sticks, demonstrating the conservation in the binding of the motifs to HA. Identical residues are labeled in black, while others are labeled in the chain color. **(E)** Sequence alignment of the IGHV6-1 germline and MEDI8852 and L5A7.5 mature heavy chains. The FG motif is highlighted in red, and yellow underline denotes regions on L5A7.5 with significant HA interactions.

To elucidate the binding interactions of L5A7 with HA we determined the crystal structure of the L5A7.5 Fab fragment as well as the cryo-EM structure of the L5A7.5 Fab bound to the HA from Indo05 (**Figure 5**, **Supplementary Figures 2, 3**, **Supplementary Tables 3, 4**). As expected, L5A7.5 bound to HA in a similar mode to MEDI8852 (**Figure 5B**). The heavy chain structures of both antibodies and their interactions with HA were nearly identical. Overlaying the HAs of the two complexes, the Fab heavy chains of L5A7.5 and MEDI8852 had an RMSD of 1.216 Å for all atoms. Remarkably, the light chains of the two antibodies, which originated from different V genes but with very similar sequences, have an RMSD of only 1.467 Å for all non-hydrogen atoms when superimposing HAs.

**Figure 6:**
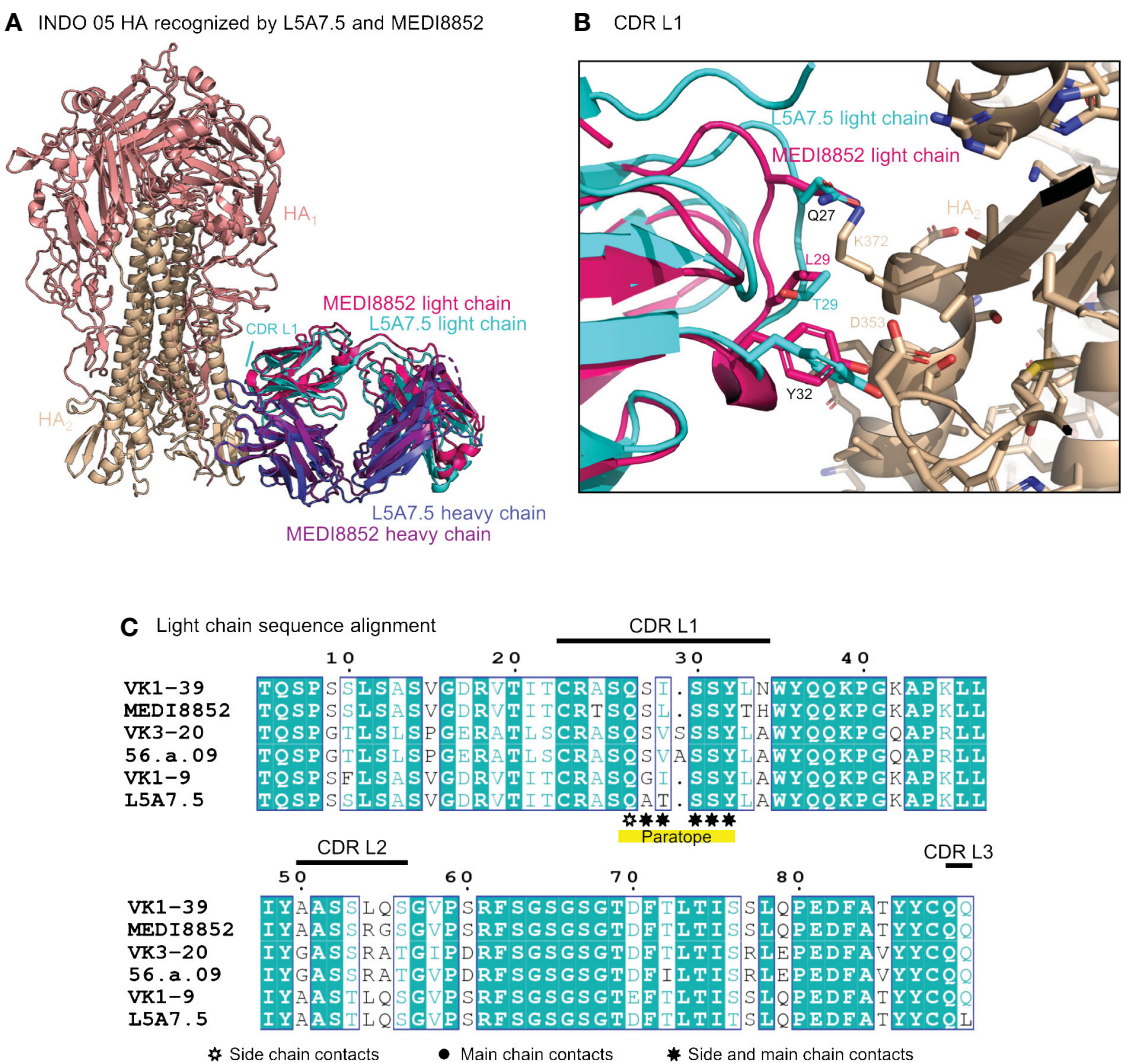
L5A7.5 utilizes a different light chain than other MEDI8852 class members, but maintains a similar sequence. **(A)** Overview of L5A7.5 binding to INDO05 HA. **(B)** Comparison of CDR L1s of MEDI8852 and L5A7.5 interacting with HA with common residues labeled in black and others in their chain color. **(C)** Sequence alignment of the light chain germline genes (IGKV1-39, IGKV3-20, IGKV1-9) and their corresponding mature antibodies (MEDI8852, 56.a.09, L5A7.5). CDRs of L5A7.5 are shown above, and the region with significant HA interaction is underlined in yellow.

Interactions between the L5A7.5 Fab and HA can be divided into three regions: (i) the FG motif residing in CDR H3, (ii) the CDR H1, and (iii) the CDR L1. In the area surrounding the FG motif, the interactions between the antibody and HA were almost perfectly conserved with MEDI8852, with both antibodies involving residues Val99_HC_ and Phe100_HC_, and either a Val100B_HC_ (MEDI8852) or Ile100B_HC_ (L5A7.5) to interact with the HA2 region of HA (**Figure 5C**). However, in the CDR H1 region, while generally similar interactions with HA were maintained, the sequences on the antibody were slightly different (**Figure 5D**). For L5A7, Phe52_HC_ had hydrophobic interactions with Val352_HA_, and Arg52B_HC_ bound to the backbone carbonyl of Gly350_HA_. However, in the case of MEDI8852, Tyr52_HC_ was responsible for both interactions. Most notably, the light chain interactions between both MEDI8852 and L5A7.5 towards HA were nearly perfectly conserved (**Figures 6A, B**), even though the two antibodies originated from different light chain genes (**Figure 6C**). Both antibodies bound through Gln27_LC_ and Tyr32_LC_, with the only difference being Leu29_LC_ in MEDI8852 versus Thr29_LC_ in L5A7.5, both having hydrophobic interaction with Lys372_HA_ side chain.

### 3.5 L5A7.5 binding to anti-idiotype 28H6E11 involves the same regions on L5A7.5 as its binding to hemagglutinin but with different chemistries

**Figure 7:**
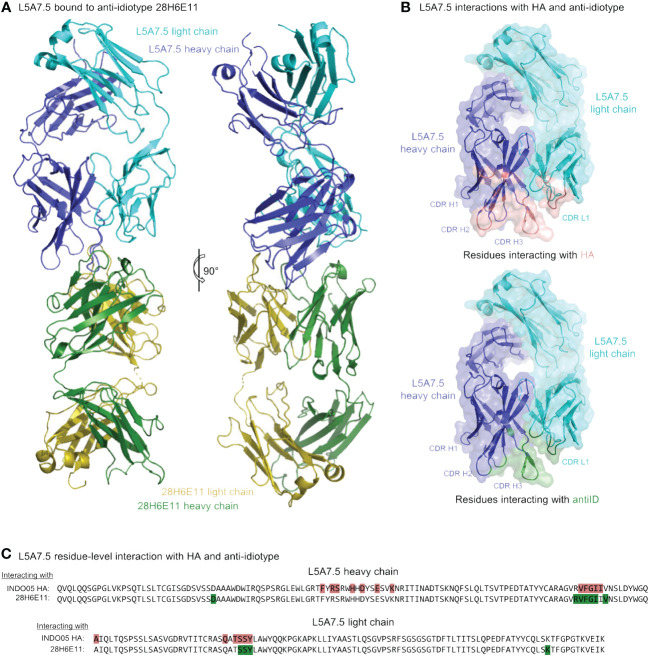
Cryo-EM structure of L5A7.5 bound to anti-idiotype reveals regions recognized by anti-idiotype to overlap with HA. **(A)** Overview of L5A7.5 bound to its anti-idiotype antibody, 28H6E11. **(B)** Binding surface on L5A7.5 which interacts with either the anti-idiotype (top) or HA (bottom), demonstrating the overlap of the two surfaces. **(C)** Sequence comparison of the interacting residues of L5A7.5 for both HA and 28H6E11. Residues with more than 25Å^2^ buried surface area at the interface are colored.

**Figure 8:**
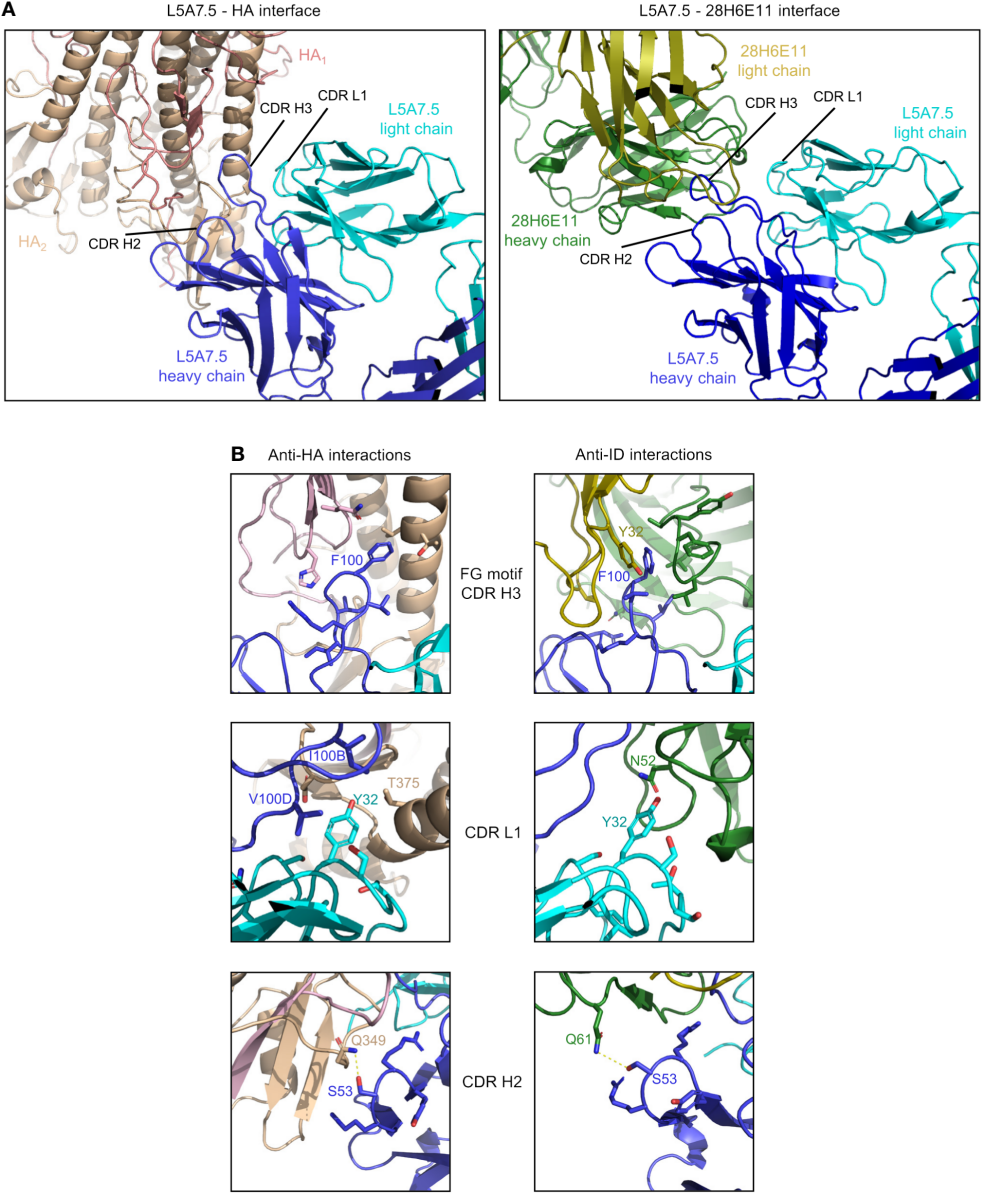
Comparison of L5A7.5 interactions with anti-idiotype antibody and HA. **(A)** Macroscopic view of the interface of L5A7.5 in complex with either HA or the 28H6E11 anti-idiotype, showing the differences in secondary structure recognized. While the epitope on HA is composed primarily of the helical regions, the corresponding epitopes on the anti-idiotype are the variable loops of the antibody. **(B)** Details of the individual interactions of L5A7.5 with HA or 28H6E11 showing the differences in interactions at the FG motif and CDR L1 interactions, while the mode of interaction at the CDR H2 is very similar.

To determine the mode of recognition of the anti-idiotype towards L5A7 lineage antibodies, we solved the structure of L5A7.5 bound to anti-idiotype 28H6E11 (**Figure 7A**, **Supplementary Figure 4**, **Supplementary Table 4**). The footprint of 28H6E11 on L5A7.5 overlapped almost completely with the paratope of L5A7.5 binding to HA, namely the CDR H1, CDR H3, and CDR L1 (**Figures 7B, C**). However, while the general interacting regions on antibody L5A7.5 were the same with both binding partners, the nature of the interactions was remarkably different. From a macroscopic view, the first major difference was in the secondary structure to which L5A7 bound (**Figure 8A**). When bound to HA, L5A7.5 primarily interacted with a helical region of HA, centered around residue Ile379_HA_ of Indo05. In contrast, when bound to the anti-idiotype antibody, L5A7.5 primarily interacted with the loops between beta strands, in particular the CDR H2, CDR H3, CDR L1 and CDR L3 of 28H6E11.

In addition to the secondary structure difference, the chemistries of a number of the interactions were substantially different between the binding to HA and anti-idiotype. For the most distinctive feature of the VH6-1 class antibodies, the FG motif in CDR H3, the anti-idiotype antibody interacted with this motif by ring stacking with residue Tyr32_antiID_ on 28H6E11 light chain, whereas in the binding to HA, the Phe100_HC_ side chain of L5A7.5 was held in a hydrophobic pocket on HA, and the backbone carbonyl of Gly100A_HC_ interacted with His32_HA_ on HA (**Figure 8B**). This difference in binding interactions resulted in a shift in the CDR H3 conformation (**Supplementary Figure 5**), reflecting the flexibility of CDR H3 in binding its targets. The L5A7.5 CDR L1 interactions with HA were dominated by the side chain of Tyr32_LC_ from L5A7.5 being held in a hydrophobic pocket formed by Val100D, Ile100B, and Thr375 on HA. However, in the interactions of L5A7.5 CDR L1 with 28H6E11, the ring OH from Tyr32_LC_ was involved in an electrostatic interaction with Asn52_antiID_. In fact, the only well conserved interaction between the two binding partners for L5A7 was in the CDR H2, in which Ser53_HC_ side chain of L5A7.5 had a hydrogen bond to the side chain of a Gln from both HA and 28H6E11 (Gln349_HA_ and Gln61_antiID_ from the heavy chain).

## 4 Discussion

In this study, we isolated VH6-1 influenza bNabs by employing anti-idiotype antibodies with specificities for sequence characteristics associated with the VH6-1 class of antibodies as capture reagents. Although antibody isolation using influenza hemagglutinin (HA) as probes has been successful in identifying numerous influenza bNabs (8, 10, 11), our strategy using anti-idiotype probes offers an additional means of selection for bNabs with specific highly desired sequence features. By using anti-idiotype antibodies 14H1F6 and 28H6E11, we identified a VH6-1 class influenza bNab lineage L5A7 capable of potently neutralizing H1, H2, H3, H5, H6, H7, H9 and H10 strains of influenza. To the best of our knowledge, this is the first reported isolation of an influenza bNab using anti-idiotype antibody reagents, and the study thus helps to validate the use of anti-idiotype reagents as a viable strategy for the isolation of bNabs.

In addition to isolating antibodies with specific features, the anti-idiotype antibodies, elicited in mice against the UCAs of VH6-1 class antibodies, can be used as probes to study VH6-1 B cell frequencies in circulation or developed as germline-targeting immunogens for the VH6-1 class. The anti-idiotype antibodies showed an appreciable degree of specificity in binding to B cells with different genetic elements associated with the VH6-1 class of bNabs in donor L16. In donor L5, however, very low frequencies of such B cells were detected. This difference likely reflects variation in VH6-1 B cell frequency in circulation. Because the blood samples from L16 and L5 used in this study were collected at random timepoints from donors whose vaccination and infection histories are likely to be different, it is possible that donors L16 and L5 had vastly different frequencies of VH6-1 class B cells in circulation at our random sampling timepoints, resulting in vastly different repertoire characteristics as measured by our anti-idiotype antibodies. Conversely, it is possible that our anti-idiotype antibodies need further optimization for improved specificity and sensitivity in binding to genetic elements of VH6-1 class B cells. We note that the combination of 14H1F6 and 28H6E11 may have been sub-optimal at reliably detecting all VH6-1 class B cells in circulation, and other combinations of our anti-idiotype antibodies may yield different results.

Despite these caveats, by using anti-idiotype antibodies 14H1F6 and 28H6E11, we were able to isolate a functionally potent and broad VH6-1 class antibody lineage – L5A7 from donor L5. The L5A7 lineage has several known sequence features associated with previously identified VH6-1 class antibodies such as IGHV6-1 gene usage, the CDR H3 “FG” motif, and CDR H3 length of 17 amino acids. It however also displays a unique feature in the usage of the IGKV1-9 gene for the light chain, a characteristic that had not been described previously for a VH6-1 influenza bNab and thereby serves to expand the definition of the influenza VH6-1 class of bNabs – and to demonstrate that anti-idiotypic antibodies can be used to select general class features.

While the general regions which L5A7 used to bind influenza A HA overlapped the region recognized by the anti-idiotype 28H6E11 antibody, the different chemistries involved in the two interactions highlight the multifaceted nature of the selection. Clearly, the selection involved features specific to VH6-1 antibodies – as demonstrated by the isolation of L5A7 – but the selected features did not involve chemical mimicry of antigen, as has generally been used in the design of vaccine immunogens. It will be interesting to see if the anti-idiotype selection used for influenza bNab identification can be adapted for anti-idiotypic vaccine elicitation. In this regard, our results suggest antibody isolation may be a useful pre-requisite for the use of anti-idiotypic antibodies as vaccine immunogens.

## Data availability statement

The isolated L5A7 Fab crystal structure presented in this study is deposited in the Protein Data Bank repository, accession number 8VVB. The cryo-EM structures of L5A7 Fab bound to INDO05 HA and of L5A7 Fab bound to 28H6E11 Fab are deposited in the Protein Data Bank repository, accession numbers 8VUE and 8VUZ, respectively, and the maps are deposited in the EMDB repository, accession numbers EMD-43529 and EMD-43545, respectively.

## Ethics statement

The studies involving humans were approved by National Institutes of Health blood bank. The studies were conducted in accordance with the local legislation and institutional requirements. The ethics committee/institutional review board waived the requirement of written informed consent for participation from the participants or the participants’ legal guardians/next of kin because Donors’ consents were waived due to anonymized donation of blood for blood donation, blood products, and research. The animal study was approved by GenScript’s Institutional Animal Care and Use Committee. The study was conducted in accordance with the local legislation and institutional requirements.

## Author contributions

AO: Investigation, Writing – original draft, Writing – review & editing. MP: Conceptualization, Investigation, Writing – original draft, Writing – review & editing. DH: Investigation, Writing – review & editing. CC: Conceptualization, Investigation, Writing – review & editing. RR: Investigation, Writing – review & editing. JG: Investigation, Writing – review & editing. AH: Investigation, Writing – review & editing. NM: Investigation, Writing – review & editing. AO: Investigation, Writing – review & editing. AS: Investigation, Writing – review & editing. SW: Writing – original draft, Writing – review & editing. WW: Investigation, Writing – review & editing. TZ: Conceptualization, Investigation, Writing – review & editing. SA: Supervision, Writing – review & editing. MK: Supervision, Writing – review & editing. LS: Supervision, Writing – review & editing. AA: Supervision, Writing – review & editing. PK: Supervision, Writing – original draft, Writing – review & editing.
