# Supplementary material for: Anti-idiotype isolation of a broad and potent influenza A virus-neutralizing human antibody
Source: Front Immunol. 2024 May 30;15:1399960. doi: 10.3389/fimmu.2024.1399960 (PMC11169713; doi:10.3389/fimmu.2024.1399960)
Supplement: Supplementary file 1 [file DataSheet_1.pdf]

Supplementary Table 1. Protein sequence of anti-idiotype antibody variable domains.

>7G1F9_HeavyChain
MEWSRVFIFLLSVTAGVHSQVQLQQSGAELVRPGTSVKVSCKASGYAFTDYLIEWIKQRPGQGLEWIGVNNPGSGETYYNEKFKDKAALTADKSSSTAFMQLSSLT
SEDSAVYFCARRFYYDYADWYFDVWGTGTTVTVSS

>7G1F9_LightChain
MKLPVRLLVLMFWIPASSSDVVMTQTPLSLPVSPGDQASISCRSSQSLVHGNGNTYLHWYLQKPGQSPKLLIYKISDRLSGVPDRFSGSGSGTDFTLNINKVEAED
LGVYFCSQSTHVPFTFGSGTKLEIK

>14H1F6_HeavyChain
MNFGLRMIFLVLTLKGVQCDVNLVESGGDLVMPGGSLILSCAASGFTFSSYTMSWVRQTPEKRLEWVATISSTGDYTYYPDIVKGRFTISRDNAKNTLYLQMSSLK
SEDTAMYYCTRVYYYGSSYWYFDVWGAGTTVTVSS

>14H1F6_LightChain
MDFQVQIFSFLLISASVIVSRGQIVLSQSPAILSASPGEKVTMTCRATSSVSYMYWYQQKPGLSPKPWIYATSKLASGVPPHFSGSGSGTSYSLTISRVEAEDAAT
YYCQQRSSNPLTFGSGTKLELK

>20C6E10_HeavyChain
MEWSRVFIFLLSVTAGVHSQVQLQQSGAELVRPGTSVKVSCKASGYAFIDYLIEWIKQRPGQGLEWIGVINPGSGETNYNEKFKGKATLTADKSSSTAYMQLSSLT
SEDSAVYFCVRRFYYDYADWYFDVWGTGTTVTVSS

>20C6E10_LightChain
MKLPVRLLVLMFWIPASSSDVVMTQIPLSLPVSLGDQASISCRSSQSLVHSNGDAYLHWYLQKPGQSPKLLIYKLSNRFSGVPDRFSGSGSGTDFTLTISRVEAED
LGVYFCSQSTHVPFTFGSGTKLEIK

>24B3D4_HeavyChain
MNFVLSLIFLALILKGVQCEVQLVESGGGLVKPGGSLKLSCTASGFTFSSFAMSWVRQTPEKRLEWVATISSVSTYTYYLDSVKGRYTISRDNAKNTLYLQMNSLG
SSDTAIYYCTRHGDYVLFYYVMDYWGQGTSVTVSS

>24B3D4_LightChain
MEKDTLLLWVLLLWVPGSTGDIVLTQSPASLAVSLGQRATISCRASESVDNSGFSFMNWFQQKPGQPPKLLIYAASNQGSGVPARFSGSGSGTDFSLNIHPMEEDD
TAMYFCQQIKEVPPTFGGGTNLEIK

>24D1C2_HeavyChain
MEWCWVFLFLLSVTAGVHSKVQLQQSGAELVKPGASVKLSCKASGYTFTDYIIHWVKQKSGQGLEWIGWFYPGSGSIKFNEKFKDKATLTADKSSSTVYLELSRLT
SEDSAVYFCARHEPGLWMDYFDYWGQGTTLTVSS

>24D1C2_LightChain
MQIISLLLISVTVIVSNGEIVLTQSPTTMASSPGDKITITCTASSSISSNYLHWYQQKPGFSPKLLIFRTSNLASGVPARFSGSGSGTSYSLTIGTLEAEDVATYF
CQEGSSIPVTFGAGTKLELK

>28H6E11_HeavyChain
MGWSCIIFFLVATATGVHSQVQLQQSGPEVVRPGVSVKISCKGSGYTFTDHAMHWVKQSHAKSLEWIGVFNTYNGDTNYNQKFEDKATMTIDKSSSTAYLELARLT
SEDSAIYYCVRLGLLGFYALDYWGQGTSVTVSS

>28H6E11_LightChain
MKLPVRLLVLMFWIPGSSSDVLMTQTPLSLPVSLGDQASISCRSSQSIVHSNGNTYLEWYLQKPGQSPKLLIYKVSNRFSGVPDRFSGSGSGTDFTLKISRVEAED
LGVYYCFQGSHVPLTFGAGTKLELK

>30F3D2_HeavyChain
MEWSRVFIFLLSVTAGVHSQVQLQQSGAELVRPGTSVKVSCKASGYAFTDYLIEWIKQRPGQGLEWIGVNNPGSGETYYNEKFKDKAALTADKSSSTAFMQLSSLT
SEDSAVYFCARRFYYDYADWYFDVWGTGTTVTVSS

>30F3D2_LightChain
MKLPVRLLVLMFWIPASSSDVVMTQTPLSLPVSPGDQASISCRSSQSLVHGNGNTYLHWYLQKPGQSPKLLIYKISDRLSGVPDRFSGSGSGTDFTLNINKVEAED
LGVYFCSQSTHVPFTFGSGTKLEIK

Supplementary Table 2. Protein sequence of L5A7 clones variable domains.

>L5A7.1_HeavyChain
QVQLQQSGPGLVKPSQTLSLTCVISGDSVSSNSAAWDWIRQSPSRGLEWLGRTYYRSKWYSDYAVSVKSRITITPDTSKNQFSLHLNSVTPEDTAVYYCARAGVRI
FGVIVDSLDYWGQGTLVTVSS

>L5A7.2_HeavyChain
QVQLQQSGPGLVKPSQTLSLTCVISGDSVSSNSAAWDWIRQTPSRGLEWLGRTYYRSKWYSDYAVSVKSRITITPDTSKNQFSLRLNSVTPEDTAVYYCARAGVRV
FGVIVDSLDYWGQGTLVTVSS

>L5A7.3_HeavyChain
QVQLQQSGPGLVKPSQTLSLTCVISGDSVSSNTAAWDWIRQSPSRGLEWLGRTYYRSRWYSDYADSVKSRITITPDTSKNQFSLHLNSVTPEDTAVYYCARAGIRI
FGIIVDSLDYWGQGTLVTVSS

>L5A7.4_HeavyChain
QVQLQQSGPRLVKPSQTLSLTCAISGDSVSSTSAAWDWIRQSSSRGLEWLGRTYYRSRWYHDYEESVKSRITINADTSKNQFSLQLASVTPEDTAVYYCARAGIRV
FGIIVDSLDYWGQGSLVTVSS

>L5A7.5_HeavyChain
QVQLQQSGPGLVKPSQTLSLTCGISGDSVSSDAAAWDWIRQSPSRGLEWLGRTFYRSRWHHDYSESVKNRITINADTSKNQFSLQLTSVTPEDTATYYCARAGVRV
FGIIVNSLDYWGQGTLVTVSS

>L5A7.1_LightChain
AIQLTQSPSSLSASVGDRVTITCRASQALNSYLAWYQQKPGKAPKLLIFAASTLQSGVPSRFSGSGSGTDFTLTISSLQPEDFATYYCQLSKTFGPGTRVDIE

>L5A7.2_LightChain
AIQLTQSPSSLSASVGDRVTITCRASQGFSSYLAWYQQKPGKAPQLLIYAASTLQSGVPSRFSGSGSGTDFTLTISSLQPEDFATYYCQQSKTFGPGTRVDIE

>L5A7.3_LightChain
AIQLTQSPSSLSASVGDRVTITCRASQGANSYLAWYQQKPGKAPKLLIYAASTLQSGVPSRFSGSGSGTDFSLTISSLQPEDFATYYCQESKTFGPGTRVDIE

>L5A7.4_LightChain
AIQLTQSPSSLSASVGDRVTITCRASQGFNSYLAWYQQKPGKAPELLIYAASTLQSGVPSRFSGSGSGTDFTLTISSLQPEDFATYYCQQSKTFGPGTKVDIK

>L5A7.5_LightChain
AIQLTQSPSSLSASVGDRVTITCRASQATSSYLAWYQQKPGKAPKLLIYAASTLQSGVPSRFSGSGSGTDFTLTITSLQPEDFATYYCQLSKTFGPGTKVEIK

Supplementary Table 3. Data collection statistics related to Supplementary Figure 1.

	**L5A7 Fab**
**PDB ID**	8VVB
**Data collection**	
Space group	$P2_12_12_1$
Cell constants	
a, b, *c* (Å)	65.760, 109.190, 140.570
a, b , g (°)	90, 90, 90
Resolution (Å)	50 – 1.73 (1.78 – 1.73)
R_{merge} (%)	8.6 (85.3)
I / s*I*	18.7 (0.8)
$CC_{1/2}$	0.999 (0.836)
Completeness (%)	94.2 (98.1)
Redundancy	11.9 (11.1)
**Refinement**	
Resolution (Å)	48 – 1.78
No. reflections	94,910 (6,197)
R_{work} / R_{free} (%)	17.38 / 20.92
No. atoms	
Protein	7722
Ligand/ion	0
Water	1193
B-factors	
Protein	23.09
Ligand/ion	N/A
Water	34.57
R.m.s. deviations	
Bond lengths (Å)	0.005
Bond angles (°)	0.845

*Values in parentheses are for the highest-resolution shell

Supplementary Table 4. Cryo-EM structure statistics related to Figures 5 and 6.

	**L5A7 Fab bound to INDO05 HA** (EMD-43529) (PDB 8VUE)	**L5A7 Fab bound to 28H6E11 Fab** (EMD-43545) (PDB 8VUZ)
**Data collection and processing**		
Magnification	105,000	105,000
Voltage (kV)	300	300
Electron exposure (e–/Å^2)	64	40
Defocus range (μm)	-0.7 to -2.0	-0.7 to -2.0
Pixel size (Å)	1.083	0.83
Symmetry imposed	C3	C1
Initial particle images (no.)	201,079	4,203,652
Final particle images (no.)	20,728	215,254
Map resolution (Å)	3.59	3.88
FSC threshold	0.143	0.143
**Refinement**		
Model resolution (Å)	3.6	3.95
FSC threshold	0.143	0.143
Model composition		
Protein residues	2724	870
Ligands	3	0
B factors (Å^2)		
Protein	69.0	52.1
Ligand	65.43	N/A
R.m.s. deviations		
Bond lengths (Å)	0.004	0.004
Bond angles (°)	0.616	0.712
Validation		
MolProbity score	1.86	2.12
Clashscore	10.27	14.49
Poor rotamers (%)	0.42	0.53
Ramachandran plot		
Favored (%)	95.30	92.81
Allowed (%)	4.70	6.96
Disallowed (%)	0.0	0.23

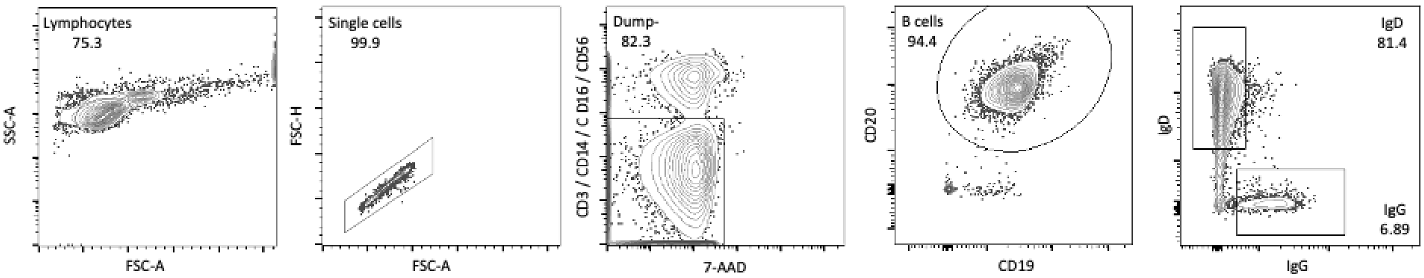

Supplementary Figure 1. Flow cytometry gating strategy. Gating scheme followed to identify naïve and memory B cells.

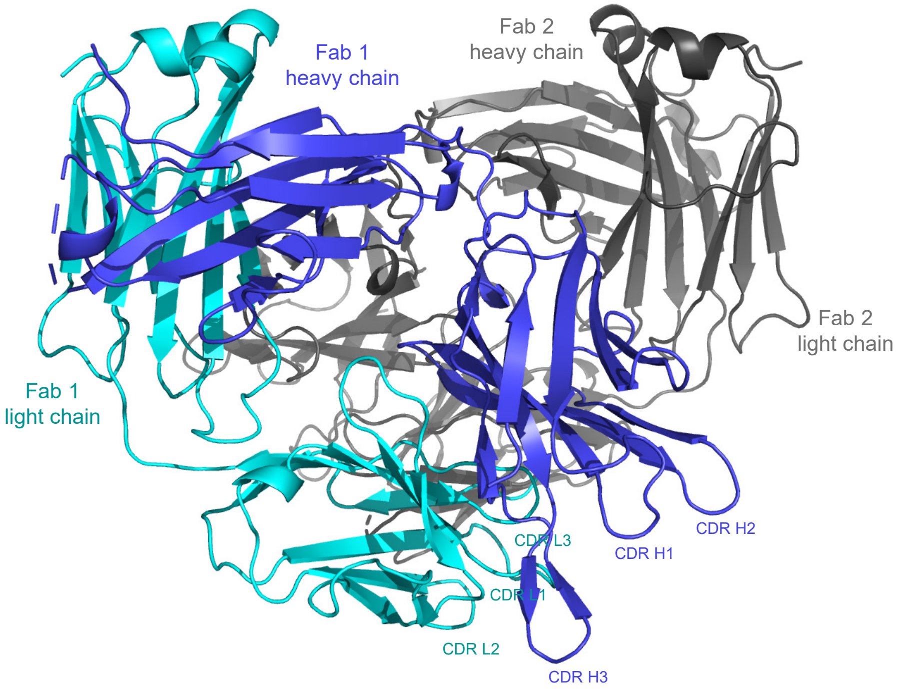

Supplementary Figure 2. Asymmetric unit of L5A7.5 Fab solved to 1.8Å. One paired heavy chain (blue) and light chain (cyan) are colored.

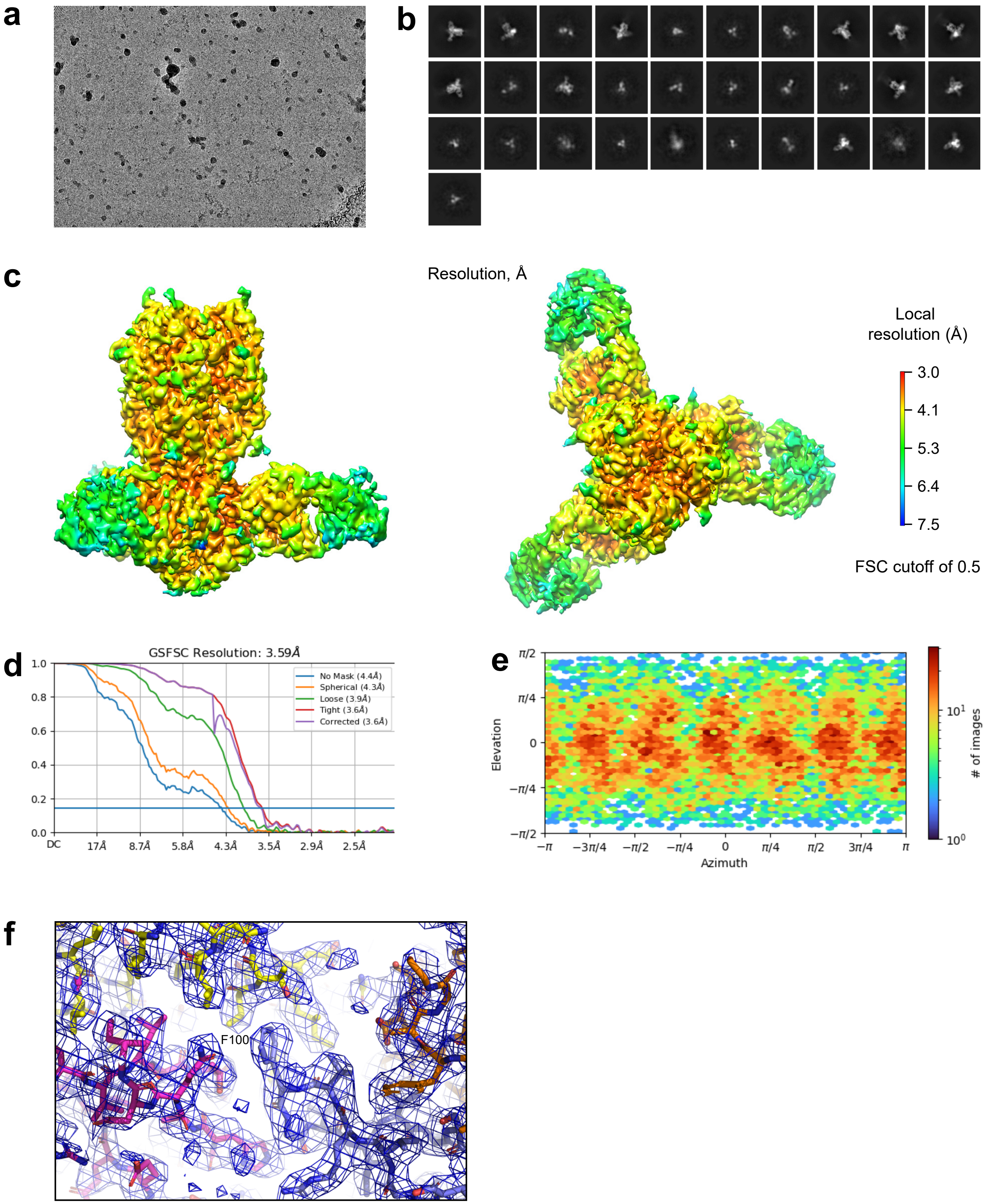

Supplementary Figure 3. Cryo-EM validation of L5A7.5-INDO05 HA complex. (**a**) Representative micrograph. (**b**) Representative 2D class averages. (**c**) The local resolution map. (**d**) The gold-standard Fourier shell correlation after non-uniform refinement. (**e**) Heatmap showing the orientations of all particles used in the non-uniform refinement. (**f**) Electron density for selected parts of the protein after focused refinement contorted at 3.0 σ in PyMOL.

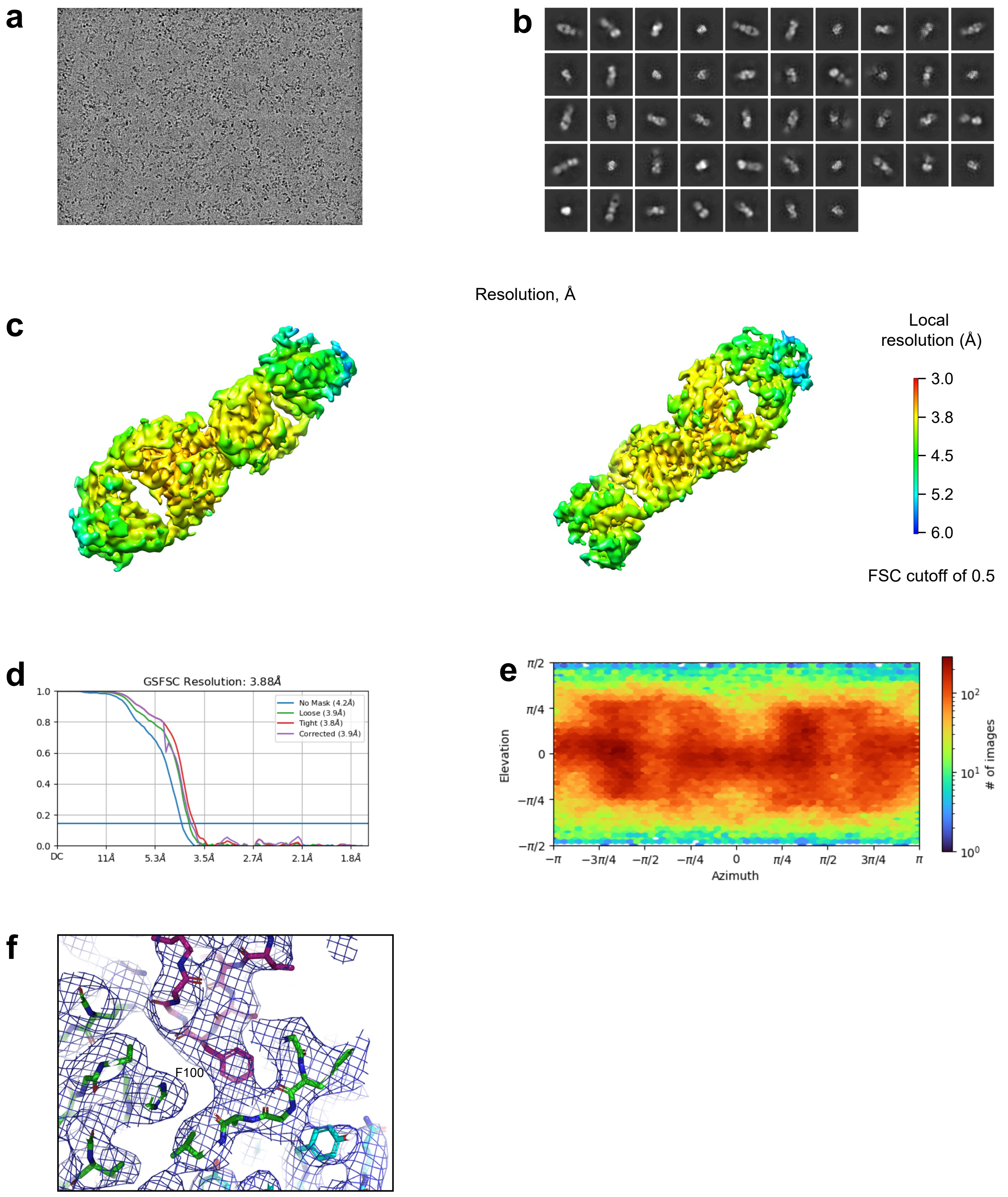

Supplementary Figure 4. Cryo-EM validation of L5A7.5-28H6E11 complex. (**a**) Representative micrograph. (**b**) Representative 2D class averages. (**c**) The local resolution map. (**d**) The gold-standard Fourier shell correlation after non-uniform refinement. (**e**) Heatmap showing the orientations of all particles used in the non-uniform refinement. (**f**) Electron density for selected parts of the protein after focused refinement contorted at 3.0 σ in PyMOL.

L5A7.5 CDR H3 movement

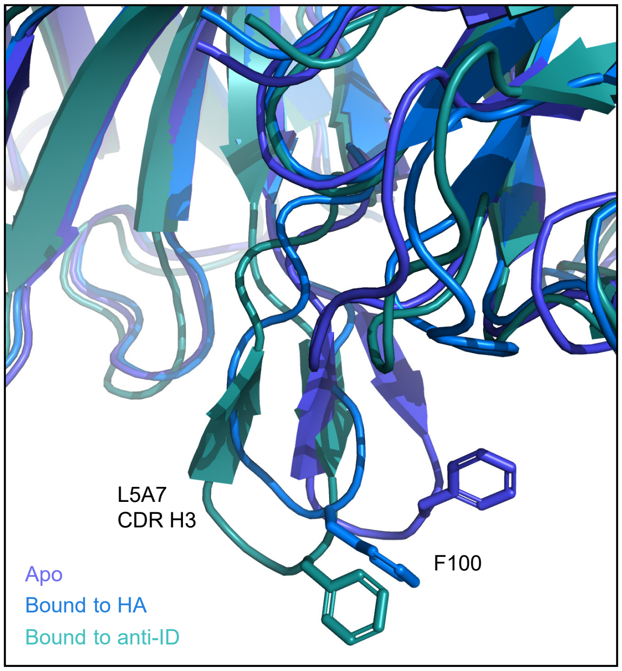

Supplementary Figure 5. L5A7.5 CDR H3 movement. View of the CDR H3 region of L5A7.5 from the apo crystal structure and the cryo-EM structures bound to HA and the anti-idiotype antibody.
